# Identification of a Novel Acid Sphingomyelinase Activity Associated with Recombinant Human Acid Ceramidase

**DOI:** 10.3390/biom13111623

**Published:** 2023-11-06

**Authors:** Xingxuan He, Edward H. Schuchman

**Affiliations:** Department of Genetics & Genomic Sciences, Icahn School of Medicine at Mount Sinai, New York, NY 10029, USA; xingxuan.he@mssm.edu

**Keywords:** ceramidase, sphingomyelinase, deacylase, Farber disease

## Abstract

Acid ceramidase (AC) is a lysosomal enzyme required to hydrolyze ceramide to sphingosine by the removal of the fatty acid moiety. An inherited deficiency in this activity results in two disorders, Farber Lipogranulomatosis and spinal muscular atrophy with myoclonic epilepsy, leading to the accumulation of ceramides and other sphingolipids in various cells and tissues. In addition to ceramide hydrolysis, several other activities have been attributed to AC, including a reverse reaction that synthesizes ceramide from free fatty acids and sphingosine, and a deacylase activity that removes fatty acids from complex lipids such as sphingomyelin and glycosphingolipids. A close association of AC with another important enzyme of sphingolipid metabolism, acid sphingomyelinase (ASM), has also been observed. Herein, we used a highly purified recombinant human AC (rhAC) and novel UPLC-based assay methods to investigate the recently described deacylase activity of rhAC against three sphingolipid substrates, sphingomyelin, galactosyl- and glucosylceramide. No deacylase activities were detected using this method, although we did unexpectedly identify a significant ASM activity using natural (C-18) and artificial (Bodipy-C12) sphingomyelin substrates as well as the ASM-specific fluorogenic substrate, hexadecanoylamino-4-methylumbelliferyl phosphorylcholine (HMU-PC). We showed that this ASM activity was not due to contaminating, hamster-derived ASM in the rhAC preparation, and that the treatment of ASM-knockout mice with rhAC significantly reduced sphingomyelin storage in the liver. However, unlike the treatment with rhASM, this did not lead to elevated ceramide or sphingosine levels.

## 1. Introduction

Acid ceramidase (AC; E.C. #3.5.1.23) is a member of the cysteine amidase family of enzymes, whose primary activity is to catalyze the removal of the fatty acid moiety from ceramide (Cer) in lysosomes, producing free fatty acids and sphingosine (Sph), the latter of which may be converted into sphingosine-1-phosphate (S1P) by the action of two distinct sphingosine kinases after transport out of lysosomes [1,2,3]. Ceramide and S1P are important signaling lipids that have varied effects in a wide range of cells, and the levels of these lipids must be carefully controlled to maintain healthy cell function and survival [4,5,6]. Numerous stress conditions, including many disease states, induce the production of Cer through the de novo synthesis or activation of sphingomyelinases, including the enzyme acid sphingomyelinase (ASM; E.C. #3.1.4.12), resulting in the breakdown of sphingomyelin (Spm) to Cer and the stimulation of the “sphingomyelin signaling pathway” [7,8]. AC activity is required to maintain healthy levels of Cer in cells and to assist in the production of sphingosine and S1P.

In mice, the complete knockout of AC function results in Cer accumulation and early embryo death between the 4- and 8-cell stage, coinciding with the onset of embryonic gene expression [9,10]. A complete knockout of AC activity has not been found in man, likely due to embryonic lethality, but partial loss-of-function mutations in the gene encoding AC (*ASAH1*) results in two severe but clinically distinct inherited disorders, Farber’s lipogranulomatosis and spinal muscular atrophy with myoclonic epilepsy [11,12,13]. Ceramide accumulation is characteristic of both disorders, although other sphingolipids may accumulate in the tissues of affected individuals as well.

Although Cer is a main substrate for AC, various data suggest that other activities may be attributed to this enzyme. For example, studies using either AC purified from mammalian tissues or recombinant human AC (rhAC) produced in Chinese hamster ovary (CHO) cells have indicated that at neutral pH, AC catalyzes a “reverse” activity that synthesizes Cer from Sph and free fatty acids [14]. In addition, it has recently been shown that at acidic pH, AC may deacylate complex sphingolipids, including galactosylceramide (GalCer), contributing to production of galactosylsphingosine (GalSph; psychosine), a neurotoxic lipid, in the brain of patients with Krabbe’s disease [15]. Other studies have shown that AC hydrolyzes bioactive *N*-acylethanolamines [16], and yet others have suggested that the beta subunit of AC may function as an Spm deacylase, producing sphingosylphosphocholine (Spc; “lyso” sphingomyelin) [17]. This latter activity competes with the endogenous sphingomyelinase activities for the Spm substrate in the skin of atopic dermatitis patients, leading to reduced levels of Spm-derived Cer, an important component of the moisture barrier required for normal skin function [18].

Other studies have also indicated a close association of AC with ASM. For example, the two enzymes may be co-precipitated from cell extracts, suggesting that they may exist within an enzyme complex [19]. Under resting conditions, AC and ASM primarily function in lysosomes, where they participate in the housekeeping functions of lipid metabolism and turnover. However, under stress conditions, they may be translocated to the outer leaflet of the plasma membrane, where they participate in cell signaling through the metabolism of sphingomyelin and ceramide. Accordingly, in healthy individuals, a small amount of ASM and AC may be detected in the circulation, but under stress conditions this increases considerably. In addition, the overexpression of a full-length human AC cDNA in CHO cells or human skin fibroblasts has been shown to lead to the overexpression of both AC and ASM activities, suggesting the potential upregulation of endogenous ASM expression in response to AC [19].

The main objective of the current study was to use a highly purified rhAC preparation to clarify the nature of the alternative activities that have been associated with AC. Overall, while we found no evidence for a deacylase activity in vitro using either Spm, GalCer or GluCer substrates, a significant ASM activity was observed using natural or fluorescent-conjugated Spm, or with the ASM-specific fluorogenic substrate, hexadecanoylamino-4-methylumbelliferyl phosphorylcholine (HMU-PC). This activity also occurred in vivo, as demonstrated by a significant reduction in Spm storage in the livers of ASM-knockout (ASMKO) mice after injection with rhAC. The rhAC-associated ASM activity was not due to contaminating CHO ASM in the rhAC preparation, as revealed by Western blotting, peptide analysis and other analytical assessments, leading to the conclusion that rhAC is responsible for both activities. The implications of these findings are discussed in this paper.

## 2. Materials and Methods

### 2.1. Reagents

Bodipy-conjugated C12 sphingomyelin (#D7711) and naphthalene-2,3-dicarboxyaldehyde (NDA) (#N-1138) were obtained from Life Technologies (Carlsbad, CA, USA). 6-Hexadecanoylamino-4-methylumbelliferyl phosphorylcholine (HMU-PC) was obtained from Biosynth (#EH31028) (Staad, Switzerland). Sphingosine (#10007907), C18 sphingomyelin (#24355), C12 ceramide (#22530), galactosylsphingosine (#31594), galactosylceramide (#24322), glucosylsphingosine (#23211), glucosylceramide (#23207) and Bodipy- (#25997) and NBD (#10007958)-conjugated C12 ceramide were obtained from Cayman Chem (Ann Arbor, MI, USA). NBD-conjugated C12 fatty acid (#790440) was obtained from Avanti Polar Lipids (Birmingham, AL, USA). Bodipy-conjugated C12 fatty acid (#D-3822) was obtained from Invitrogen (Waltham, MA, USA). All other biochemical reagents were obtained from Thermo Fisher Scientific (Hampton, NH, USA).

### 2.2. Animal Studies

The ASMKO mouse colony was established from heterozygous breeding pairs as described previously [20]. All experiments were performed at the Icahn School of Medicine under a protocol (#98-0089) approved by the Institutional Animal Care and Use Committee and adhered to the NIH guidelines for the use of experimental animals. Animals were fed food and water ad libitum, and euthanasia was performed by ketamine/xylazine injections followed by cervical dislocation according to NIH guidelines. For the treatment studies, 4-month-old ASMKO mice were randomly divided into two groups (8 mice/group): (1) rhAC treatment group: ASMKO mice were injected intraperitoneally with 10 mg/kg rhAC; (2) recombinant human ASM (rhASM) treatment group: ASMKO mice were injected intraperitoneally with 1 mg/kg rhASM. For each treatment group, a control group was included, where ASMKO mice were injected with the vehicle alone (8 mice/group). All mice were injected every other day for six injections, and 24 h after the last injection, the mice were euthanized, and the livers were collected for lipid analysis. The rhASM used in this study was produced and purified from CHO cell media as previously described [21].

### 2.3. rhAC Production and Purification

Human recombinant AC (rhAC) was produced in CHO cells as previously described [19]. Briefly, a full-length human AC cDNA was inserted into a mammalian expression vector and transfected into the dihydrofolate-reductase-negative DG44 CHO cell line. Stably transfected clones were isolated and expanded, and one cell clone (CHO6) was selected based on its high AC activity. The CHO6 cells were then expanded and initially grown in DMEM supplemented with 10% *v*/*v* FBS and 1% penicillin/streptomycin. Over time, the FBS concentration was reduced to 1%, and the low-serum, conditioned medium containing rhAC was collected and concentrated by pressure filtration (cut-off 30 kDa, Amicon, Billerica, MA, USA). rhAC was then purified using a fast protein liquid chromatography (FPLC) system with three columns (Concanavalin A Sepharose, Blue Sepharose, and Superose 12 Gel) (Amersham Biosciences, Piscataway, NJ, USA).

### 2.4. AC Activity Assay

AC activity was routinely determined using fluorescently (NBD) conjugated C12-Cer as previously described [22]. Briefly, samples were incubated at 37 °C (1:1, *v*/*v*) with substrate buffer (0.2 mM NBD-C12 Cer, 0.2 M citrate/phosphate buffer, pH 4.5, 0.3 M NaCl, 10% FBS and 0.2% Igepal) for 30 min. The reaction was stopped by ethanol (10×) and centrifuged (13,000× *g*/10 min), and the supernatant (5 μL) was then analyzed using an Acquity H-Class UPLC system (Waters Corporation, Milford, MA, USA). Separation of the undegraded NBD-C12 Cer substrate and NBD-C12 fatty acid reaction product was achieved using a BEH C18 column (2.0 × 30 mm, 1.7 μm). The fluorescent product (NBD-C12 fatty acid) was monitored at excitation and emission wavelengths of 435 nm and 525 nm, respectively. Quantification of the product peak was calculated using the Waters Empower software (version Empower 3 Pro) according to a standard curve derived from commercial NBD-C12 fatty acid. Alternatively, in some experiments, AC activity was determined using unlabeled C12-Cer as the substrate and the same buffer/reaction conditions as described above for NBD-C12 Cer. The Sph product produced in this reaction was quantified after derivatization with NDA using a Waters Acquity H-Class UPLC system (see below).

### 2.5. ASM Activity Assay

ASM activity was routinely determined using fluorescently (Bodipy) conjugated C12-Spm as previously described [23]. Briefly, samples were mixed with an ASM assay buffer (0.2 mM Bodipy-C12 Spm, 0.2 M sodium acetate buffer, pH 5.0, 0.2 mM ZnCl_2_). After 30 min of incubation at 37 °C, the reactions were stopped by ethanol (10×) and centrifuged (13,000× *g*/10 min), and the supernatant (5 μL) was analyzed using an Acquity H-Class UPLC system (Waters). Separation of the Bodipy-C12 Spm substrate and Bodipy-C12 Cer product was achieved using a BEH-amide 1.7 μm column (2.1 mm × 50 mm) and monitored at excitation and emission wavelengths of 500 and 520 nm, respectively. ASM activity was determined based on quantitation of the area under the peak of the product, Bodipy-C12 Cer, according to a standard curve prepared using a commercial standard. ASM activity was also determined using the fluorogenic substrate, HMU-PC, as previously described [24] or by assessing the hydrolysis of unlabeled C18-Spm.

### 2.6. AC Deacylase Activity Assay

A novel, fluorescence-based assay method was developed to determine the potential deacylase activity of rhAC using three substrates (Spm, GalCer and GalCer). Reaction mixtures (100 mM potassium acetate buffer (pH 4.5), sodium acetate buffer (pH 6.5) or HEPES buffer (pH 7.2) containing 20 mM CaCl_2_, 1μg/μL rhAC, 0.2% Igepal and 0.1 mM of the respective substrate) were incubated at 37 °C for up to 24 h, and they were then subjected to NDA derivatization for 10 min at 50 °C [25] followed by centrifugation (13,000× *g*) for 10 min. The derivatized products (NDA-Spc, NDA-GalSph or NDA-GluSph, respectively) were then separated and quantified using a Waters Acquity UPLC system as described below for the quantification of Sph.

### 2.7. Quantification of Tissue Sphingomyelin, Ceramide and Sphingosine

Lipid extracts were prepared from liver homogenates by the classic Folch method [26] using chloroform/methanol (2:1). The lipid extract was then dried under nitrogen gas and re-dissolved in a 2% Igepal solution, and Spm, Cer and Sph levels were measured as previously described [27,28]. Briefly, to determine the total Spm content in the sample, the 2% Igepal lipid extract was re-suspended in a hydrolysis buffer containing rhASM and rhAC. This completely hydrolyzed the Spm in the extract to Cer, and then the Cer to Sph. The Sph was then derivatized into a fluorescent product by reaction with the fluorogenic compound, NDA, and analyzed using an Acquity H-Class UPLC system (Waters) monitored at excitation and emission wavelengths of 252 and 483 nm, respectively. Quantification of the fluorescent Sph was calculated using the Waters Empower software according to a standard curve derived from commercial Sph. For the quantification of Cer, the same procedure was used except that the Spm hydrolysis step was excluded (i.e., only rhAC was added to the 2% Igepal lipid extract). For the quantification of Sph, the same procedure was used except that both hydrolysis steps were excluded (i.e., no rhASM or rhAC were added to the lipid extract). In this way, the endogenous Sph present in the lipid extracts could be determined directly. After derivatization and quantification, the net Spm content in the sample was determined by taking the total Spm value and subtracting the total Cer value. The net Cer content was determined by taking the total Cer value and subtracting the endogenous Sph value.

### 2.8. Gel Staining with Coomassie Blue and Silver Nitrate

Purified enzyme samples were treated in Laemmli sample buffer (Bio-Rad Lab Inc., Hercules, CA, USA) at 90 °C for 10 min, loaded onto 4–20% gradient mini-protean TGX gels (Bio-Rad Lab Inc., Hercules, CA, USA) and electrophoresed in a mini-protean Tetra system (Bio-Rad Lab Inc., Hercules, CA, USA) at 200 V for 30 min. Following electrophoresis, the gels were stained using either Sampleable SafeStain (Invitrogen, Waltham, MA, USA) or a Pierce Silver Stain Kit (Thermo Fisher Scientific, Hampton, NH, USA) according to the manufacturer’s instructions.

### 2.9. Peptide Analysis

Purified rhAC (100 μg) was denatured with 7.0 M guanidine-HCl, 50 mM Tris and pH 7.5, and the denatured protein was subjected to reduction with 10 mM DTT at room temperature for 30 min followed by alkylation with 20 mM iodoacetamide for 20 min, in the dark, at room temperature. The alkylation was quenched with 4 μL of 50 mM DTT. The guanidine-HCl was then diluted to a final concentration of 1 M. Half of the sample was digested overnight with trypsin/LysC (Promega, #V5072) at a ratio of 1:10 (protein/enzyme; *w*:*w*) and the other half was digested overnight with Asp-N (Promega, #V1621) at a ratio of 1:25 (protein/enzyme; *w*:*w*) at 37 °C. Separation of the peptides was accomplished using a Waters UPLC system fitted with a Waters CSH C18 column (150 × 2.1 mm, 1.7 μm, 130 Å) at 50 °C. Mobile phase A consisted of 0.1% formic acid in water, while mobile phase B consisted of 0.1% formic acid in acetonitrile. A total of 2 μg of the trypsin/LysC digested sample and 4 μg of AspN digested sample were injected and separated by UPLC, and the peptide sequences were then determined by mass spectrometry with an SCIEX X500B QTOF system fitted with a Turbo VTM ion source with a twin sprayer probe using information-dependent acquisition (IDA) for the 8 most abundant ions.

### 2.10. Western Blot Analysis

Samples were separated by SDS-PAGE using 4–20% gradient mini-protean TGX gels under reducing conditions and Tris/glycine/SDS buffer (Bio-Rad Lab Inc., Hercules, CA, USA), and they were then transferred onto 0.2 μm PVDF membranes (Bio-Rad Lab Inc., Hercules, CA, USA) using a semidry Trans-Blot Turbo transfer system (Bio-Rad Lab Inc., Hercules, CA, USA). For immunoblot analysis, membranes were blocked with PBS/Tween-20 containing 5% dry milk and then incubated with the first antibodies: (1) rabbit anti-human AC polyclonal antibody produced by Covance/LabCorp using rhAC provided by us (Princeton, NJ, USA) or (2) rabbit anti-human ASM polyclonal antibody (Thermo Fisher Scientific, Hampton, NH, USA, #PA577047). Both primary antibodies were recognized by the goat anti-rabbit secondary antibody IRDye 800CW (LI-COR, Lincoln, NE #926-3221). Detection was achieved using an ODYSSEY CLx scanner and the IS Studio software (version IS studio 5.2.5) (LI-COR, Lincoln, NE, USA). For quantitative analysis, the total intensity of the bands was assessed using the ImageJ software (version ImageJ 1.48). Quantification was calculated by multiplying the area of the band by the average intensity (average number of pixels/area).

### 2.11. Statistical Analyses

For two-group comparisons, the Mann–Whitney U-test for non-parametric data or a two-sample Student’s *t*-test for data with parametric distribution was used. For multiple comparisons, data with a normal distribution were analyzed by one-way ANOVA followed by a Tukey post hoc test. The statistical significance of the non-parametric data was determined by the Kruskal–Wallis test to analyze all experimental groups. *p*-values (*p*) < 0.05 were considered significant, and the values are indicated on the individual figures. The GraphPad Prism 6.0 software (GraphPad Software, La Jolla, CA, USA) was used for all statistical analyses.

## 3. Results

### 3.1. Purification of rhAC

**Figure 1:**
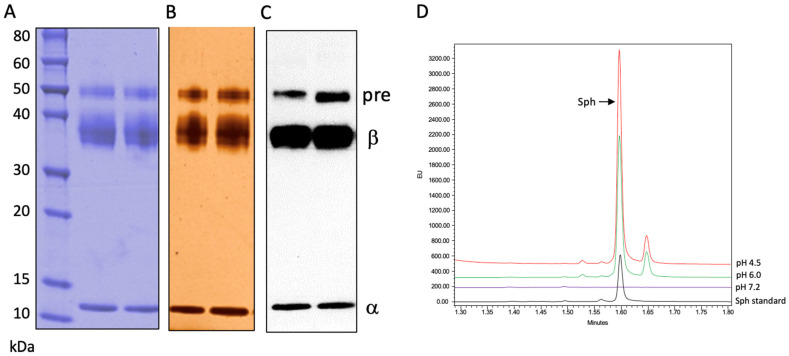
rhAC was purified from the media of overexpressing CHO cells and run on SDS-PAGE-reducing gels as described in the Methods. Three AC polypeptides (precursor, pre; beta, β and alpha, α) were identified by (**A**) Coomassie blue staining, (**B**) silver staining and (**C**) Western blotting using an anti-human AC polyclonal antibody. A total of 100 μg of rhAC was loaded per lane. (**D**) Ceramidase activity was determined using C12-Cer as the substrate at three different pH values. The Sph product was derivatized with NDA as described in the Methods, and the fluorescent Sph was identified and quantified by UPLC. The experiments were repeated three times.

Figure 1A,B show the Coomassie blue and silver staining of the purified rhAC. Three AC polypeptides were identified under reducing conditions: the ~50 kDa precursor, ~40 kDa beta and ~13 kDa alpha subunits. Each of these polypeptides were recognized by an anti-human AC polyclonal antibody after Western blotting (Figure 1C). Peptide sequencing performed after digestion with two different proteases (Trypsin/Lys-C and Asp-N) revealed only AC-related peptides in the preparation, and the purified rhAC exhibited a high ceramidase activity at acidic pH towards the unlabeled C12-Cer substrate (Figure 1D).

### 3.2. In Vitro Evaluation of rhAC Deacylase Activity towards Galactosyl- and Glucosylceramide

**Figure 2:**
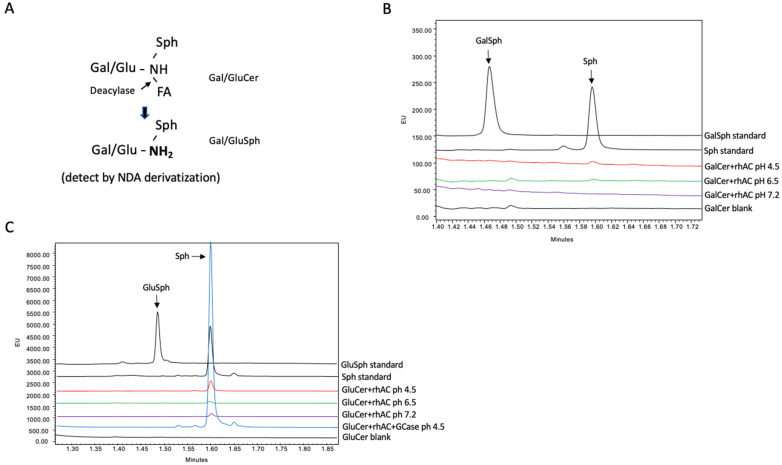
The deacylase activity of rhAC was analyzed using either GalCer or GluCer as substrates. (**A**) Schematic depiction of the deacylation reaction. The GalSph or GluSph products were derivatized with NDA and quantified by UPLC as described in the Methods. Reactions were incubated for up to 24 h at 37 °C prior to analysis. Note that no GalSph (**B**) or GluSph (**C**) products were produced in these reactions under three different pH conditions. For GluCer, an additional control reaction was included, in which GluCer was first incubated with the enzyme beta glucocerebrosidase (GCase) followed by rhAC. In this combined reaction, GluCer was first hydrolyzed to Cer by GCase, and then Cer was hydrolyzed to Sph by rhAC. The Sph product was derivatized with NDA and quantified by UPLC. Note the significant Sph product produced by the dual enzyme reaction at pH 4.5, confirming the high AC activity associated with rhAC. The experiments were repeated three times.

A UPLC method was developed to determine the potential deacylase activity of rhAC towards the glycosphingolipid substrates, GalCer and GluCer. This method was based on the NDA derivatization procedure commonly used to detect Sph [27], adapted to detect the GalSph and GluSph products of the deacylation reactions, respectively (see schematic, Figure 2A). As shown in Figure 2B,C, no GalSph or GluSph products were found under the three different pH conditions when the reactions were carried out using purified rhAC as the enzyme source and either GalCer or GluCer as the substrates. As a control, we added the enzyme glucocerebrosidase (GCase) to the reaction mixture with GluCer as the substrate, and an Sph peak was observed at acidic pH, resulting from the hydrolysis of GluCer to Cer by GCase followed by the hydrolysis of the Cer product to Sph by rhAC.

### 3.3. In Vitro Evaluation of rhAC Deacylase Activity towards Sphingomyelin and Identification of a Novel Acid Sphingomyelinase Activity

**Figure 3:**
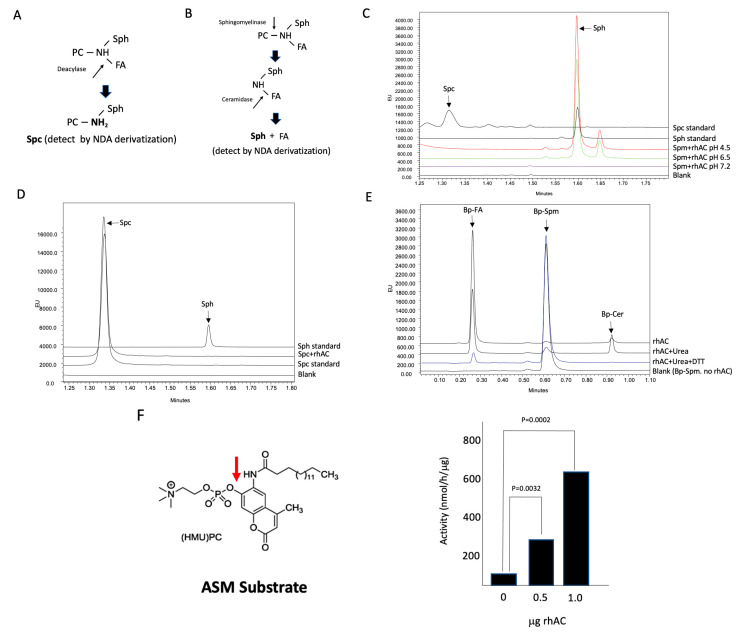
Evaluation of the Spm deacylation reaction using rhAC and identification of a novel rhAC-associated ASM activity using three different substrates. (**A**) Schematic depiction showing the potential deacylase reaction using C18-Spm. Detection of the Spc product after derivatization with NDA and quantification by UPLC was performed as described in the Methods. (**B**) Schematic depiction showing that a potential combined reaction of sphingomyelinase activity followed by ceramidase activity of C18-Spm would result in Sph, which could be detected and quantified using NDA. (**C**) Deacylation reactions were carried out for 24 h with C18-Spm as the substrate, but no Spc product was detected under three different pH conditions. However, a significant Sph product was detected at pH 4.5, indicating the presence of an ASM activity. (**D**) To ensure that Spc could not be directly hydrolyzed to Sph by rhAC, we incubated rhAC with Spc, but no Sph product was found. To further confirm the rhAC-associated ASM activity, we incubated rhAC with Bodipy-conjugated C12-Spm (Bp-Spm) at pH 4.5 and found a high ASM activity (Bp-FA product) that was sensitive to denaturation using urea and DTT (**E**). (**F**) As an additional confirmation of the rhAC-associated ASM activity, we used the ASM-specific fluorogenic substrate HMU-PC and found significant ASM activity in rhAC. The experiments were repeated three times. *p* values are shown in (**F**) comparing the activities with varying amounts of rhAC added to the reaction mixtures to the buffer controls. The mean and standard deviation values were 52.5 +/− 9.92, 297.35 +/− 16.1 and 617 +/− 27.1 for buffer alone (0 rhAC added), 0.5 and 1.0 μg/rhAC, respectively.

We next adapted the NDA/UPLC detection method to determine whether rhAC could deacylate C18-Spm to C18-Spc (see schematic, Figure 3A). As shown in Figure 3C, we did not observe an Spc product in these reactions at any pH, but we surprisingly did detect a very significant Sph product under acidic conditions. We reasoned that this likely occurred from the combined hydrolysis of Spm to Cer (ASM activity), followed by Cer hydrolysis to Sph (AC activity) (see schematic, Figure 3B). To rule out the possibility that the Sph was derived from the direct hydrolysis of Spc, we next tested our rhAC preparation using Spc as a substrate, but we found no Sph product (Figure 3D). To further test whether rhAC had an associated ASM activity, we used Bodipy (Bp)-conjugated C12-Spm as the substrate, and found a significant Bp-conjugated fatty acid product in these reactions under acidic conditions, derived from the combined hydrolysis of Spm to Cer followed by the hydrolysis of Cer to Sph and fatty acid (Figure 3E). This reaction was partially inhibited with urea, and it was almost completely inhibited with urea and DTT, indicating that it was dependent on the rhAC protein present in the reaction mixtures. In the presence of urea, a small Bp-Cer peak also was observed, consistent with the partial inhibition. Lastly, we used the ASM-specific fluorogenic substrate HMU-PC to assess the ASM activity, and we confirmed a high activity using rhAC as the enzyme source (Figure 3F). In this reaction, fluorescence is only generated if the phosphocholine moiety is removed from the HMU backbone.

**Figure 4:**
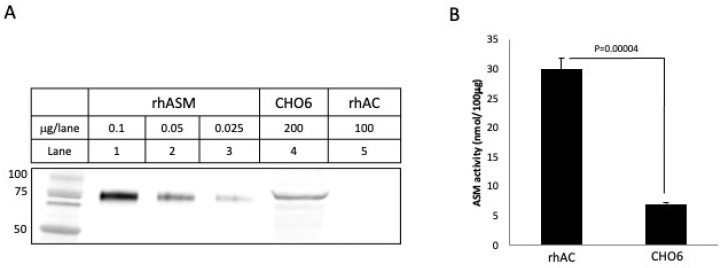
To further rule out the possibility that the rhAC-associated ASM activity could be derived from contaminating CHO ASM, we performed Western blot analysis using an anti-ASM antibody that cross-reacted with CHO ASM (**A**). Note that CHO6 cell extracts overexpressing rhAC had a cross-reacting CHO ASM band that was readily visible. In contrast, the highly purified rhAC did not. We then compared the ASM activity in purified rhAC vs. CHO6 cells, and despite the fact that no cross-reacting CHO ASM was detected by Western blot in rhAC, the ASM activity was >5 fold greater than that in CHO6 cell extracts (**B**). This demonstrated that the rhAC-associated ASM activity could not be derived from CHO ASM. The experiments were repeated three times. The mean ASM activity value for rhAC in (**B**) was 29.9, with a standard deviation of +/−2.0, while for the CHO6 cells, it was 7.0 +/− 0.136. *p* values are shown.

Thus, we concluded from the above experiments that an unexpected, but significant, ASM activity was present in the highly purified rhAC preparation, despite the fact that no ASM-related peptides were observed in the preparation and no evidence of CHO-derived ASM was observed after Coomassie blue or silver staining. To further rule out the possibility of contaminating CHO ASM in our rhAC preparation, we also performed Western blotting with a polyclonal anti-human ASM antibody that cross-reacted with the CHO ASM. As shown in Figure 4A, this antibody could detect as little as 0.025 μg of purified rhASM after Western blotting, and it also identified the CHO-derived ASM in the CHO6 cell extracts expressing rhAC. No CHO-derived ASM was identified using this antibody in our highly purified rhAC preparation, even when 100 μg of the purified rhAC was loaded on the gels. We then compared the ASM activity in the CHO6 cell extracts to that in rhAC (Figure 4B) and found a >5-fold higher activity in the rhAC preparation, despite the lack of ASM cross-reacting material. Based on the Western blotting results, if this ASM activity was derived from contaminating CHO ASM in our preparation, it should have been readily detected, but it was not.

### 3.4. In Vivo Evaluation of the rhAC-Associated Acid Sphingomyelinase Activity

**Figure 5:**
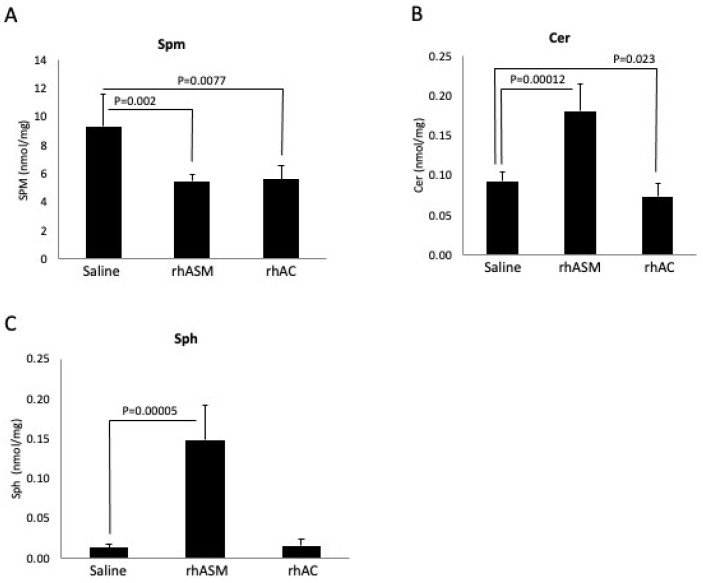
Four-month-old ASMKO mice accumulating high levels of Spm in their livers were treated with 6 injections of rhASM (1 mg/kg) or rhAC (10 mg/kg). A total of 24 h after the last injection, the mice were euthanized, and the livers were collected for Spm, Cer or Sph analysis as described in the Methods. Previous studies have shown that treatment of ASMKO mice with 10 mg/kg rhASM leads to acute toxicity [29], including lethargy and death. No toxicity was observed using 10 mg/kg rhAC, despite the equivalent level of Spm reduction by the two enzyme treatments (**A**). As expected, treatment with rhASM led to Cer and Sph elevations (**B**,**C**). However, despite the equivalent reduction in Spm in the ASMKO mouse livers, no Cer or Sph elevation was observed after rhAC treatment, consistent with the fact that dosing of these mice with 10 mg/kg of rhAC did not lead to toxicity. The experiments were repeated three times. In (**A**), the mean SPM values and standard deviations are 9.34 +/− 2.22, 5.50 +/− 0.482 and 5.64 +/− 0.910 for saline, rhAC and rhAC, respectively; in (**B**), the mean Cer values and standard deviations are 0.093 +/− 0.011, 0.181 +/− 0.033 and 0.074 +/− 0.016, respectively; and in (**C**), the mean Sph values are 0.014 +/− 0.004, 0.148 +/− 0.044 and 0.015 +/− 0.009, respectively. *p* values are shown.

The in vitro experiments above indicated that the rhAC had a significant ASM activity, and that this was not derived from contaminating CHO ASM. To further confirm this activity, we carried out a study where ASMKO mice were treated with either rhASM (1 mg/kg) or rhAC (10 mg/kg) by six intraperitoneal injections every other day and euthanized 24 h following the last injection. Based on previous work [29], 1 mg/kg is the highest initial dose of rhASM that can be used in these mice without toxicity. Surprisingly, we found that the accumulated Spm in the ASMKO mouse livers was reduced to a similar degree by either enzyme treatment (Figure 5A). However, while the rhASM treatment led to an expected Cer and Sph elevation (Figure 5B,C), there was no accumulation of Cer or Sph in the livers of the mice treated with rhAC. In accordance with these results, no treatment-related toxicity was observed in the ASMKO mice treated with rhAC at 10 mg/kg, in contrast to previous observations where an equivalent dose of rhASM led to inflammation, lethargy and death [29]. The treatment of ASMKO mice with 1 mg/kg rhAC led to modest, but insignificant, reductions in liver Spm (not shown).

## 4. Discussion

Acid ceramidase is a key regulator of ceramide signaling and sphingolipid metabolism and, as such, is subject to careful genetic and post-translational regulation. For example, it is synthesized as an inactive precursor protein and must undergo self-cleavage into alpha and beta subunits, as well as N-glycosylation, to obtain full ceramidase activity [30]. The self-cleavage reaction is promoted by low pH, but other factors are likely to influence it as well. Intriguingly, at physiologic pH, AC does not hydrolyze Cer efficiently, but rather it can synthesize it using Sph and free fatty acids as substrates [14]. This “reverse” activity has been observed in vitro using purified preparations of mammalian AC and in situ following the expression of the human AC cDNA in cultured cells. It has been further shown that AC may carry out deacylation reactions by removing fatty acids from complex sphingolipid substrates such as Spm and glycosphingolipids, consistent with a broader amidase capacity and substrate recognition [15,16,17,18]. For example, it has been shown that the beta subunit of AC catalyzes the deacylation of Spm and that this activity is elevated in atopic dermatitis, contributing to low Cer levels and disruption of the skin’s water barrier. Other studies have indicated that the enzyme may deacylate GalCer and GluCer, resulting in the “lyso” forms of these lipids (GalSph and GluSph, respectively). In the lysosomal storage disorder, Krabbe’s disease, GalSph (psychosine) is an important neurotoxic lipid, and based on genetic studies using knockout mice, it has been suggested that inhibitors of AC might be used as therapeutic agents to prevent GalSph build-up in these patients [15].

Thus, we initiated the current study with the goal of confirming whether a highly purified rhAC preparation made in our laboratory could perform these deacylation reactions in vitro. To examine this, we developed a novel UPLC-based assay method, in which the deacylation products of Spm, GalCer and GluCer (Spc, GalSph and GluSph, respectively) could be separated and quantified following NDA derivatization. NDA is a fluorescent reagent that reacts with free amino groups and is commonly used to quantify Sph [27,31]. Because free amino groups are also present in these deacylation products, we reasoned that the same derivatization reaction could be used to visualize and quantify these lipids as well.

After confirming that the NDA reaction could be used to detect these lipids and developing methods to separate and quantify them by UPLC, we evaluated the ability of purified rhAC to deacylate Spm, GalCer and GluCer under various pH conditions. However, in contrast to previous studies, we found no Spc, GalSph or GluSph products, even after long (24 h) incubation periods. The rhAC used in these studies was purified from the media of overexpressing CHO cells and is a unique mixture of AC precursor, alpha and beta polypeptides, associated through disulfide bonds. It is highly purified and has a high AC activity towards natural and fluorescently conjugated Cer substrates, but it is distinct from the endogenous AC. Therefore, although we did not detect any deacylase activities in this enzyme preparation, we cannot rule out the possibility that these reactions may occur under different conditions or with different AC preparations.

Surprisingly, in the course of our experiments evaluating the deacylase reaction using Spm as a substrate, we found a high sphingomyelinase activity present in the rhAC preparation. This activity was detected using natural (C-18 Spm) and artificial (Bodipy-conjugated C12 Spm) substrates, and it was sensitive to denaturing and reducing agents, indicating that it was inherent to the protein. It was also sensitive to pH, with the optimal activity detected at pH 4.5. The rhAC-associated ASM activity was further detected in vivo, where we found an equivalent reduction in Spm in the liver of ASMKO mice using 1 mg/kg rhASM vs. 10 mg/kg rhAC. Notably, it has been previously shown that treatment of ASMKO mice with 10 mg/kg rhASM leads to lethargy, acute inflammation and death [29], but this did not occur using 10 mg/kg rhAC. Dosing of ASMKO mice with 1 mg/kg rhAC also led to Spm reduction, although this was only about 20% of that obtained using purified rhASM.

In order to rule out the possibility that a small amount of contaminating CHO-derived ASM was responsible for this rhAC-associated ASM activity, we carried out two experiments. First, we performed Western blotting using a polyclonal anti-ASM antibody that cross-reacted with CHO ASM (Figure 4). No CHO-derived ASM was detected in the rhAC preparation, despite the high ASM activity. In addition, after digestion with trypsin/LysC or Asp-N and analysis by mass spectrometry, only AC peptides were found. These data, together with the results of the Coomassie blue and silver staining, demonstrated that no contaminating CHO-derived ASM was present in the rhAC preparation.

Notably, the in vivo studies also revealed that while rhAC had an intrinsic ASM activity, it was not as efficient as endogenous ASM. This was evident from two observations. First, the ASMKO mice, which contain an endogenous AC, still accumulate Spm. Second, the injection of 10 mg/kg rhAC into the ASMKO mice led to an Spm reduction equivalent to that of 1 mg/kg rhASM. This reduced biologic activity of the AC-associated vs. endogenous ASM activity could be due to reduced Spm binding and/or catalytic activity, or potentially due to differential cellular uptake and delivery of the rhAC vs. rhASM to the proper subcellular compartment. Another intriguing observation was that high-dose (10 mg/kg) treatment with rhAC did not cause toxicity in the ASMKO mice, while the treatment with the same dose of rhASM did [29]. To further understand this observation, we evaluated the levels of the downstream Spm hydrolysis products, Cer and Sph, in the livers of the treated mice. Although the Cer and Sph levels were elevated in the livers of the ASMKO mice treated with rhASM (1 mg/kg), there was no elevation of these lipids in the animals treated with rhAC, even at a dose of 10 mg/kg and despite the fact that the Spm levels were reduced to the same degree. These observations are consistent with previous studies using rhAC to treat mice with AC deficiency [22]. Although these mice accumulated massive amounts of Cer in their livers that was significantly reduced by the rhAC treatment, they never exhibited Sph elevation or toxicity, even using doses of up to 10 mg/kg.

The differential impact of rhAC and rhASM on downstream lipid metabolism is important and might explain the differential impact on toxicity as well. In the case of rhASM, the Spm is hydrolyzed to Cer, and then the endogenous AC is required to hydrolyze the Cer further to Sph. In the case of rhAC, we propose that the same enzyme is responsible for both Spm and Cer hydrolysis. Although both reactions produce Sph, it is possible that they act in different cell compartments with different access to sphingosine kinases or other downstream metabolic enzymes. A further understanding of the differential metabolism of Sph in rhASM- vs. rhAC-treated mice is an important area for future studies and could have implications for understanding the high-dose toxicity associated with rhASM treatment [29,32].

These findings also have important implications for understanding Farber’s disease and SMA-PME. Although both diseases are caused by a deficiency in AC activity, they have notably different phenotypes that cannot be simply explained by the levels of residual AC activity. Indeed, even within the Farber disease phenotype, the spectrum of disease is remarkable, ranging from an early onset disease that involves the central nervous system (CNS) to later onset forms that are often misdiagnosed as idiopathic arthritis. In the case of SMA-PME, the disease is mostly limited to the CNS and includes progressive muscle weakness and seizures. Based on the multiple activities attributed to AC, including the new findings reported here regarding the rhAC-associated ASM activity, it is possible that mutations in the gene encoding AC (*ASAH1*) could have differential effects on these AC activities, leading to variable and complex levels of substrate accumulation and thus clinical presentation.

In conclusion, we identified a significant ASM activity associated with highly purified rhAC and studied this reaction in vitro and in vivo. We ruled out the possibility that the rhAC-associated ASM activity was due to contaminating CHO-derived ASM and further demonstrated this activity in ASMKO mice, where despite significant Spm degradation, this activity did not elicit Cer or Sph accumulation or cause high-dose toxicity. We also reported a new in vitro method to detect the deacylase activity of rhAC by NDA derivatization, although when using this method, we did not find any deacylation reaction products using Spm, GalCer and GluCer as substrates. Understanding the biological role of this novel AC-associated ASM activity will be the subject of future investigations.

## 5. Conclusions

We unexpectedly found ASM activity in a highly purified preparation of rhAC. We ruled out the possibility that this activity could be derived from contaminating CHO ASM, and we further showed that after injection into ASMKO mice, this rhAC-associated ASM could significantly reduce Spm storage in the liver without causing high-dose toxicity. These findings have important implications for our understanding of the AC-deficiency diseases, Farber’s disease and SMA-PME, as well as the high-dose toxicity associated with enzyme replacement therapy for acid-sphingomyelinase-deficient Niemann–Pick disease (ASMD). They also reveal a new activity attributed to rhAC that further highlights its complex and central role in sphingolipid metabolism.

## Data Availability

All original data generated from this study are available upon request.
